# Histologic Response to Induction Chemotherapy in High‐Risk Neuroblastoma

**DOI:** 10.1002/cam4.71332

**Published:** 2025-11-26

**Authors:** Monica Pomaville, Pei‐Chi Kao, Antonio Perez‐Atayde, Wendy B. London, Rani E. George

**Affiliations:** ^1^ Department of Pediatric Oncology Children's Hospital of Philadelphia Philadelphia Pennsylvania USA; ^2^ Department of Pediatrics Harvard Medical School Boston Massachusetts USA; ^3^ Boston Children's Cancer and Blood Disorders Center Boston Massachusetts USA; ^4^ Department of Pathology Boston Children's Hospital Boston Massachusetts USA; ^5^ Department of Pediatric Oncology Dana‐Farber Cancer Institute Boston Massachusetts USA

**Keywords:** histology, neuroblastoma, prognosis, risk stratification

## Abstract

**Introduction:**

Tumor histology at diagnosis is used in conjunction with other prognostic features to risk stratify patients with neuroblastoma and to assign therapy regimens. In patients with high‐risk disease, adjustment of therapy is tailored to treatment response, largely based on disease imaging following induction chemotherapy and resection of the primary tumor. The goals of this study were to (i) quantify changes in histologic features in the primary tumor between diagnosis and resection, and (ii) assess the prognostic capability of such alterations.

**Methods:**

Tumor histology from paired samples at diagnosis and resection was evaluated from 94 patients with high‐risk neuroblastoma enrolled in Children's Oncology Group (COG) trials from 2001 to 2013. Presence of Schwannian stroma, neuropil, degree of differentiation, mitosis karyorrhexis index (MKI), necrosis, and percentage of neuroblastic cells were annotated. Changes in tumor histology between diagnosis and resection were analyzed for association with overall survival (OS) and progression‐free survival (PFS).

**Results:**

Significant changes between diagnosis and resection were observed in all histologic parameters (*p* < 0.01), suggesting a more phenotypically differentiated tumor following induction therapy. A higher percentage of intermediate‐high MKI in tumor cells at diagnosis was associated with a lower PFS and OS (*p* < 0.05). No other histologic factor was associated with survival at diagnosis or resection. The tumor percentage of intermediate‐high MKI decreased by a mean of 75% from diagnosis to resection (*p* < 0.0001). The shift from intermediate/high MKI at diagnosis to low MKI at resection had a PFS hazard ratio (HR) of 2.1 (95% CI: 0.9, 4.9; *p* = 0.0908) and OS HR = 2.3 (95% CI: 0.9, 5.9; *p* = 0.0773).

**Conclusion:**

Our findings suggest that primary neuroblastoma tumors undergo a significant morphologic shift following induction chemotherapy to a differentiated, less mitotically active phenotype. However, the alterations are not prognostic of patient outcome either at resection alone, or when the change from diagnosis to resection is considered.

## 1 Introduction

Neuroblastoma is a tumor of the sympathetic nervous system originating from the neural crest and manifesting in the adrenal glands or paraspinal ganglia. It is the most commonly diagnosed malignancy in the first year of life, accounting for 8% to 10% of all childhood cancers and approximately 15% of deaths due to cancer [1]. While patients classified as having low‐ or intermediate‐risk disease exhibit an overall survival rate of over 95%, outcomes for patients with high‐risk disease remain poor [2]. The diagnosis of neuroblastoma is based mainly on histopathological confirmation from either the primary tumor site or bone marrow infiltration. Treatment, however, is informed by risk stratification, based on a combination of clinical and biological prognostic factors such as age, stage, tumor histology and *MYCN* status [3, 4]. Age over 18 months, tumor *MYCN* amplification, unfavorable histology, segmental chromosome aberrations, and variations in tumor cell ploidy are characteristic of high‐risk disease [2, 5].

The first pathological system for neuroblastoma was established in 1984, in which the assessment of Schwannian stroma, tumor cell differentiation status, MKI, presence of nodules, and age were used to classify tumors as having either favorable or unfavorable histology [6, 7]. Initially coined the Shimada system, this classification evolved into the International Neuroblastoma Pathology Classification (INPC) [8]. The INPC relied on a more comprehensive set of features to differentiate between favorable and unfavorable histology that included diagnostic category, MKI, and age. The diagnostic category was expanded to include ganglioneuroma, ganglioneuroblastoma (intermixed), ganglioneuroblastoma (nodular), and neuroblastoma (undifferentiated, poorly differentiated, and differentiating subtypes). The International Neuroblastoma Risk Group (INRG) task force, formed in 2004, incorporated biological annotations together with tumor histology into risk stratification and generated the INRG risk classification, a system similar to that used by the Children's Oncology Group (COG), with the aim of centralizing neuroblastoma risk stratification internationally [2, 9].

The current standard of care in the US for patients with newly diagnosed high‐risk neuroblastoma starts with a tissue diagnosis, followed by up to 6 cycles of induction chemotherapy, consisting primarily of vincristine, cisplatin, etoposide, cyclophosphamide, topotecan, and doxorubicin [10]. Primary tumor resection typically occurs after the fourth or fifth cycle of induction chemotherapy, with subsequent administration of local radiation, myeloablative therapy with stem cell rescue, differentiation therapy with retinoic acid and immunotherapy with the anti‐GD2 antibody, dinutuximab [3, 4, 11]. Risk stratification at diagnosis has, for the most part, enabled the selection of patients most likely to benefit from this regimen [3, 12]. However, there remains a group of high‐risk patients who are unresponsive to standard therapy or who experience relapse [13]. Optimization of response assessment early in and throughout treatment could allow for the identification of patients who could be assigned to alternative therapies. This strategy could achieve the objective of minimizing long‐term side effects by avoiding more cytotoxic treatment in those who respond well, or alternatively, modifying therapy in those who do not respond.

Several parameters have been evaluated for response prediction during treatment [14]. The 1993 International Neuroblastoma Response Criteria (INRC) was established to assess clinical response to induction chemotherapy and revised in 2017 to incorporate modern imaging modalities [15]. Response to treatment is evaluated by quantitative m‐iodobenzylguanidine (mIBG) scoring [16, 17, 18] and cross‐sectional imaging as part of Response Evaluation Criteria in Solid Tumors (RECIST) criteria [19]. mIBG as a response assessment after induction therapy has been prognostic in multiple analyses; however not all tumors are mIBG‐avid [16, 20]. For non‐MIBG avid tumors, [^18^F] fluorodeoxyglucose (FDG)—positron emission tomography (PET) has been used; however this signal can be non‐specific and is not reflective of functional tumor [19]. Reduction in primary tumor size with cross‐sectional imaging using non‐standard volumetric assessment was prognostic in one study; however, it was no longer significant in patients who received dinutuximab therapy [21, 22]. Urine catecholamine levels are not considered predictive of outcome due to the influence of outside factors on their levels [23]. Individual histologic characteristics of the tumor at diagnosis have been shown to be independently prognostic [24]. We had earlier reported that in a limited group of patients, intermediate/high tumor MKI and presence of ≥ 90% persistent tumor cells in the primary tumor following induction chemotherapy are prognostic of early relapse and poor overall survival [25]. Our results suggested that alterations in tumor histology in response to induction chemotherapy, as well as the degree of such change, may be predictive of outcome. We therefore attempted to validate our initial findings in a larger cohort of patients.

## 2 Methods

### 2.1 Patients

This retrospective analysis was approved by the Institutional Review Boards of Dana‐Farber Cancer Institute and Boston Children's Hospital. Specimen approval was obtained from COG. Tumor samples were obtained from patients who were enrolled in COG ANBL00B1 and various therapeutic trials between 2001 and 2013. Eligibility criteria included a diagnosis of high‐risk neuroblastoma at diagnosis and the availability of matched tissue samples at diagnosis and primary tumor resection following induction chemotherapy. Patients had to have experienced an event (relapse, progression, death from any cause) or be followed for a minimum of 2 years without an event.

### 2.2 Histopathological Review

Tumor sections obtained at diagnosis and resection were retrospectively reviewed by three of the authors (A.P.‐A., R.E.G., M.P.) who were blinded to the risk classification, biology, and outcome of the patient. Standard procedures had been used to prepare hematoxylin–eosin (H&E) sections after paraffin embedding of tumor tissue fixed in 10% buffered formaldehyde. Slides with inadequate preparation were excluded from the analysis. Between one and seven tissue sections from each matched tumor pair at diagnosis and resection were reviewed. Slides were scored based on several morphological features that were part of the INPC [8, 26] (Table 1). Based on the amount of Schwannian stroma, tumors were classified as stroma‐poor or stroma‐rich. Tumor differentiation was scored based on the proportion of differentiating neuroblasts and presence of neuropil as undifferentiated (no signs of maturation, no differentiated neuroblasts, or neuropil), poorly differentiated (neuropil present but < 5% ganglionic differentiation), or differentiating (> 5% ganglionic differentiation) [8, 26, 27, 28]. MKI was determined based on the proportion of cells undergoing active mitosis or karyorrhexis as low (< 2%), intermediate (2%–4%), or high (> 4%). The amount of neuropil was classified as absent/minimal, moderate, or abundant. In addition, in the resected specimens, the percentage of (i) tumor necrosis (defined as areas with necrosis, hemorrhage, fibrosis, calcification, granulation tissue, histiocyte replacement and/or hemosiderin deposition) and (ii) persistent neuroblastic cells (NCs) (areas showing undifferentiated, poorly differentiated, or differentiating NCs, but without ganglioneuroblastoma (GNB) or ganglioneuromatous elements) relative to the total tumor section on each slide, were assessed. Tumors were divided into GNB, intermixed (stroma‐rich tumors with microscopic well‐defined nests of neuroblasts at different stages of maturation) or GNB, nodular (macroscopically visible nodules of stroma‐poor tumor tissue in stroma‐rich or stroma‐dominant tissues) [26].

**TABLE 1:** Demographic characteristics of patients in study (*n* = 94).

Continuous characteristics	Median (range)
Age at diagnosis (years)	3.1 (0.1–22.7)

Categorical characteristics	*n* (%)
INSS stage	
Stage 2A	1 (1.1%)
Stage 3	9 (9.6%)
Stage 4	82 (87.2%)
Stage 4S	2 (2.1%)
*MYCN* status	
Amplified	36/83 (43.4%)
Not amplified	47/83 (56.6%)
Unknown	11
Histology	
Favorable	2/86 (2.3%)
Unfavorable	84/86 (97.7%)
Unknown	8

### 2.3 Statistical Analysis

Histologic features were analyzed as either continuous or categorical variables. For continuous variables (NCs, differentiation status, necrosis), the average percentage of tumor was determined for each patient, at diagnosis and at resection. Analyses were performed of the continuous value as well as a binary value, by dichotomizing using a cutoff, such as a > 10% threshold for tumor necrosis. For categorical variables, binary variables were created by combining categories consistent with previous reports (stroma‐poor and stroma‐rich, low MKI and intermediate‐high MKI) [25]. Since patients had multiple tissue sections at a given timepoint, the classification associated with the poorest outcome for that timepoint (stroma‐poor, intermediate‐high MKI, ≤ 10% necrosis) was identified. Then, the proportion of sections with that poorest outcome classification was determined, and the average proportion across sections at diagnosis, and resection was determined. Histologic features were descriptively summarized. We tested for significant differences between diagnosis (D) versus resection (R). For continuous variables, a Wilcoxon signed‐rank test was used to identify statistically significant changes from diagnosis to resection. The mean (±standard deviation) difference between diagnosis and resection (R − D) was calculated across patients. For categorical variables, McNemar's test was used to identify statistically significant changes from diagnosis to resection in the proportion of patients with each histologic characteristic. Analyses were repeated within the subgroup of patients > 18 months of age at diagnosis with *MYCN* non‐amplified disease [29].

OS was defined as the time from diagnosis to death, censored on the date of last contact. PFS was defined as the time from diagnosis to the first occurrence of an event (relapse, progression, or death), censored on the date of last contact without an event. Survival curves were generated using the methods of Kaplan and Meier with standard errors according to Greenwood. Individual histopathological features (sections classified as stroma‐poor, MKI intermediate‐high, minimal neuropil, NCs, differentiation, ganglioneuroma, necrosis) were tested for prognostic ability in univariate Cox proportional hazards models of OS and PFS. A sample size of *n* = 94 was calculated as sufficient to detect a 32% difference in 3‐year PFS using a one‐sided log‐rank test with 80% power and alpha = 0.05.

## 3 Results

### 3.1 Patient Characteristics

Pathological material was available for analysis from 94 patients, the majority of whom were treated with COG‐based induction therapy protocols (Table 1). Ages ranged from 7.5 weeks to 22 years at diagnosis, with a median age of 3 years. The majority (82/94; 87.2%) of samples were from patients with INSS stage 4 disease, of which 47 had *MYCN* non‐amplified tumors and were aged ≥ 18 months. Tumor *MYCN* amplification was present in 36 (43.4%) patients. All but two patients were classed as having unfavorable tumor histology at diagnosis as per COG review. The median follow‐up time without an event was 9.8 years (range: 2.1–14.7 years).

### 3.2 Changes in Histologic Characteristics Following Induction Therapy

There were 94 diagnostic specimens, although the numbers of evaluable paired tumors for each histologic parameter varied due to quality and type (e.g., tumor‐infiltrated bone marrow samples) (Table 2). The vast majority of tumors at diagnosis had unfavorable histology—stroma poor (90%) with undifferentiated or poorly differentiated cells (94%), having high or intermediate MKI (87%) [8]. A statistically significant change from diagnosis to resection i.e., following induction chemotherapy was observed for these histological features (*p* < 0.0001) and for every other feature assessed, including the amount of neuropil and stroma (Table 2). The characteristic with the greatest difference between diagnosis and resection was MKI, whereby the tumor percentage of intermediate/high MKI decreased by a mean of 75% (*p* < 0.0001) from diagnosis to resection (Table 2). Other histological characteristics that were found to be significantly decreased from diagnosis to resection when analyzed as continuous variables included undifferentiated or poorly differentiated status (−41.7, *p* < 0.0001) and persistent neuroblastic cells ≥ 90% (−38.3%, *p* < 0.0001). Interestingly, the proportion of differentiating neuroblastoma cells decreased significantly at resection (−38.7%, *p* < 0.0001), although a small proportion of tumors showed evolution into ganglioneuroma. Moreover, tumor necrosis was increased (30.3%, *p* < 0.0001), with the proportion of tumors with necrosis > 10% increasing from diagnosis to resection (37.4%, *p* < 0.0001) (Table 2). Analyzed categorically, similar trends were seen in tumor histology between diagnosis and resection for the following features: significant decreases in the proportion of tumors with intermediate/high MKIs, undifferentiated or poorly differentiated status and ≥ 90% NCs (*p* < 0.0001). Samples with ≤ 10% necrosis also decreased significantly implying that there was a significant increase in tumor necrosis following induction (−26%, *p* < 0.0001) (Table 2). Overall, following induction therapy, the histological changes suggested a switch to a more differentiated tumor phenotype.

**TABLE 2:** Histologic factors at diagnosis and resection, and identification of significant changes from diagnosis to resection (*n* = 94).

Histologic factor (continuous)^a^	Timepoint	Paired sample size *n* (%)	Mean percentage of tumor	*p* *
Stroma poor	Diagnosis (D)	85 (90)	99.4	< 0.0001
	Resection (R)		85.2	
	(R − D)		−14.2	
MKI Int/High	Diagnosis (D)	82 (87)	81.8	< 0.0001
	Resection (R)		6.8	
	(R − D)		−75.0	
Neuropil—minimal	Diagnosis (D)	88 (94)	54.3	0.01
	Resection (R)		33.9	
	(R − D)		−20.4	
NCs	Diagnosis (D)	94 (100)	84.4	< 0.0001
	Resection (R)		46.0	
	(R − D)		−38.3	
NCs ≥ 90%	Diagnosis (D)	94 (100)	70.4	< 0.0001
	Resection (R)		24.1	
	(R − D)		−46.2	
Differentiation—UD/PD	Diagnosis (D)	88 (94)	97.9	< 0.0001
	Resection (R)		56.2	
	(R − D)		−41.7	
Differentiating	Diagnosis (D)	88 (94)	82.8	< 0.0001
	Resection (R)		44.2	
	(R − D)		−38.7	
Ganglioneuroma	Diagnosis (D)	94 (100)	0.05	< 0.0001
	Resection (R)		8.1	
	(R − D)		8.06	
GNB, inter/nod	Diagnosis (D)	1 (1)	100	ND
	Resection (R)		100	
	(R − D)		0	
Necrosis (%)	Diagnosis (D)	94 (100)	15.6	< 0.0001
	Resection (R)		45.9	
	(R − D)		30.3	
Necrosis > 10% (%)	Diagnosis (D)	94 (100)	29.6	< 0.0001
	Resection (R)		67.0	
	(R − D)		37.4	

Histologic factor (binary)^b^	Timepoint	Sample size *n* (%)	*n* (%)	*p* **
Stroma poor	Diagnosis (D)	85 (90)	85 (100)	0.008
	Resection (R)		77 (91)	
	(R − D)		−8 (−9)	
MKI Int/High	Diagnosis (D)	82 (87)	70 (85)	< 0.0001
	Resection (R)		9 (11)	
	(R − D)		−61 (−74)	
NCs ≥ 90%	Diagnosis (D)	94 (100)	72 (77)	< 0.0001
	Resection (R)		38 (40)	
	(R − D)		−34 (−36)	
Differentiation—UD/PD	Diagnosis (D)	88 (94)	87 (99)	< 0.0001
	Resection (R)		66 (75)	
	(R − D)		−21 (−24)	
GNB, inter/nod	Diagnosis (D)	1 (1)	1 (100)	ND
	Resection (R)		1 (100)	
	(R − D)		0 (0)	
Necrosis ≤ 10%	Diagnosis (D)	94 (100)	72 (77)	< 0.0001
	Resection (R)		48 (51)	
	(R − D)		−24 (−26)	

Abbreviations: GNB, ganglioneuroblastoma; NCs, neuroblastic cells.

^a^ Factors with continuous values were assessed as mean percentage of the whole tumor sample across sections, at each timepoint.

^b^ Histologic factors with binary values were created by combining categories or applying a percentage threshold.

* Wilcoxon signed‐rank test comparing diagnosis versus resection.

** McNemar's test of paired comparison of diagnosis versus resection.

### 3.3 Prognostic Capabilities of Histologic Features at Diagnosis and Resection

At diagnosis, stroma, neuropil, differentiation status, or necrosis showed no prognostic value when analyzed as categorical variables. Patients with intermediate/high tumor MKI at diagnosis had poorer outcomes compared to those with low tumor MKI (5‐year PFS: 32% ± 5.6% vs. 50% ± 14.4%, *p* = 0.0895; 5‐year OS: 43% ± 6.0% vs. 67% ± 13.6%, *p* = 0.0731), although this difference was not statistically significant (Table 3). However, a higher percentage of intermediate/high MKI tumor content at diagnosis was associated with lower PFS and OS. In other words, for every 1% increase in the proportion of intermediate‐high MKI tumor content at diagnosis, the risk of a PFS event increased by 1% [HR = 1.01, 95% CI: (1.001–1.02); *p* = 0.0462], and the risk of an OS event increased by 1% [HR = 1.01, 95% CI: (1.001–1.02); *p* = 0.0392] (Table 4). When assessed at tumor resection, the percentage of tumors with intermediate/high MKI was no longer associated with PFS [HR = 0.998, 95% CI: (0.99–1.01); *p* = 0.6991] or OS [HR = 0.999, 95% CI: (0.99–1.01); *p* = 0.8732]. Status of stroma, neuropil, NCs, differentiation, or necrosis was also not prognostic of PFS or OS at the time of tumor resection (Tables 3 and 4).

**TABLE 3:** Prognostic ability of categorical histologic factors at diagnosis, at resection, and the change from diagnosis to resection (*n* = 94).

Histologic factor (categorical)	Timepoint	*n*	5‐year PFS ± SE (%)	PFS hazard ratio (95% CI)^a^	PFS *p*‐value*	5‐year OS ± SE (%)	OS hazard ratio (95% CI)^a^	OS *p*‐value*
Stroma		85						
Poor	Diagnosis (D)	85	37 ± 5.3	ND	ND	49 ± 5.5	ND	ND
Rich [ref]		0	ND			ND		
Poor	Resection (R)	77	36 ± 5.5	1.4 (0.6, 3.6)	0.4438	46 ± 5.8	1.3 (0.5, 3.1)	0.6617
Rich [ref]		8	50 ± 17.7			75 ± 15.3		
Poor → Rich	(D → R)	8	50 ± 17.7	[ref]		75 ± 15.3	[ref]	
Poor → Poor		77	36 ± 5.5	1.4 (0.6, 3.6)	0.4438	46 ± 5.8	1.3 (0.5, 3.1)	0.6617
Rich → Rich		0	ND	ND	ND	ND	ND	ND
Rich → Poor		0	ND	ND	ND	ND	ND	ND
MKI		82						
Int/High	Diagnosis (D)	70	32 ± 5.6	2.1 (0.9, 4.9)	0.0895	43 ± 6.0	2.3 (0.9, 5.9)	0.0731
Low [ref]		12	50 ± 14.4			67 ± 13.6		
Int/High	Resection (R)	9	33 ± 15.7	1.1 (0.5, 2.6)	0.8113	44 ± 16.6	1.2 (0.5, 2.8)	0.6728
Low [ref]		73	35 ± 5.6			47 ± 5.9		
Low → Low	(D → R)	12	50 ± 14.4	[ref]		67 ± 13.6	[ref]	
Int/High → Low		61	32 ± 6.0	2.1 (0.9, 4.9)	0.0908	43 ± 6.4	2.3 (0.9, 5.9)	0.0773
Int/High → Int/High		9	33 ± 15.7	2.0 (0.7, 6.4)	0.2155	44 ± 16.6	2.4 (0.7, 8.0)	0.1437
Low → Int/High		0	ND	ND	ND	ND	ND	ND
Differentiation		88						
UD/PD	Diagnosis (D)	87	35 ± 5.2	ND	ND	47 ± 5.4	ND	ND
Differentiating [ref]		1	1.0 ± 0			1.0 ± 0		
UD/PD	Resection (R)	66	38 ± 6.0	0.9 (0.5, 1.5)	0.6056	46 ± 6.2	0.95 (0.5, 1.8)	0.8797
Differentiating [ref]		22	30 ± 10.1			54 ± 10.8		
UD/PD → UD/PD	(D → R)	65	37 ± 6.0	[ref]		46 ± 6.2	[ref]	
D'ing → UD/PD		1	1.0 ± 0	0 (0, 0)	0.9895	1.0 ± 0	0 (0, 0)	0.9848
UD/PD → D'ing		22	30 ± 10.1	1.1 (0.6, 2.0)	0.6642	54 ± 10.8	1.02 (0.6, 1.9)	0.9412
D'ing → D'ing		0		ND	ND	ND	ND	ND
Histology category		1						
GNB, inter/nod	Diagnosis (D)	1	0 ± 0	ND	ND	0 ± 0	ND	ND
GN [ref]		0	ND			ND		
GNB, inter/nod	Resection (R)	1	0 ± 0	ND	ND	0 ± 0	ND	ND
GN [ref]		0	ND			ND		
GNB, inter/nod → GNB, inter/nod	(D → R)	1	0 ± 0	[ref]		0 ± 0	[ref]	
GN → GN		0	ND	ND	ND	ND	ND	ND
GNB, inter/nod → GN		0	ND	ND	ND	ND	ND	ND
GN → GNB, inter/nod		0	ND	ND	ND	ND	ND	ND
NCs category		94						
≥ 90%	Resection (R)	38	33 ± 7.8	1.1 (0.7, 1.9)	0.6536	37 ± 8.1	1.3 (0.8, 2.2)	0.3330
< 90% [ref]		56	39 ± 6.5			57 ± 6.6		
Necrosis category		94						
≤ 10%	Resection (R)	48	37 ± 7.1	0.95 (0.6, 1.6)	0.8419	44 ± 7.3	1.2 (0.7, 2.0)	0.5725
> 10% [ref]		46	37 ± 7.1			54 ± 7.4		

Abbreviations: D'ing, differentiating; ND, not done; [ref], reference category.

^a^ Amount of increased risk for every one‐unit increase of the value of the histologic factor.

* Univariate Cox PH model.

**TABLE 4:** Prognostic ability of continuous histologic factors for PFS and OS, at diagnosis, at resection, and the change from diagnosis to resection.

Histologic factor (continuous)	Timepoint	*n* ^a^	PFS hazard ratio (95% CI)^b^	PFS *p*‐value*	OS hazard ratio (95% CI)^b^	OS *p*‐value**
Stroma poor (%)	Diagnosis (D)	85	0.98 (0.94, 1.02)	0.3523	0.98 (0.94, 1.02)	0.2448
	Resection (R)		1.01 (0.996, 1.01)	0.2529	1.003 (0.99, 1.01)	0.5286
	(R − D)		1.01 (0.997, 1.01)	0.2139	1.003 (0.995, 1.01)	0.4475
MKI Int/High (%)	Diagnosis (D)	82	1.01 (1.0002, 1.02)	**0.0462** **	1.01 (1.001, 1.02)	**0.0392** **
	Resection (R)		0.998 (0.99, 1.01)	0.6991	0.999 (0.99, 1.01)	0.8732
	(R − D)		0.99 (0.99, 1.00)	0.0506	0.99 (0.99, 1.00)	0.0588
Neuropil—minimal (%)	Diagnosis (D)	88	0.998 (0.99, 1.00)	0.3854	0.998 (0.99, 1.00)	0.5748
	Resection (R)		0.997 (0.99, 1.00)	0.3682	0.999 (0.99, 1.01)	0.7256
	(R − D)		1.0 (0.996, 1.00)	0.9476	1.0 (0.996, 1.01)	0.8349
NCs (%)	Diagnosis (D)	94	0.998 (0.989, 1.01)	0.6470	0.997 (0.99, 1.01)	0.5670
	Resection (R)		1.004 (0.997, 1.01)	0.2553	1.01 (0.998, 1.01)	0.1879
	(R − D)		1.004 (0.998, 1.01)	0.2035	1.01 (0.998, 1.01)	0.1366
Differentiation—UD/PD (%)	Diagnosis (D)	88	1.02 (0.98, 1.07)	0.2624	1.02 (0.98, 1.07)	0.2747
	Resection (R)		0.999 (0.99, 1.01)	0.6903	1.0 (0.99, 1.01)	0.9745
	(R − D)		0.997 (0.99, 1.00)	0.4022	0.999 (0.99, 1.01)	0.6415
Differentiation (%)	Diagnosis (D)	88	0.999 (0.99, 1.01)	0.8789	0.998 (0.99, 1.01)	0.6240
	Resection (R)		1.001 (0.99, 1.01)	0.7334	1.001 (0.99, 1.01)	0.7874
	(R − D)		1.001 (0.995, 1.01)	0.6896	1.002 (0.995, 1.01)	0.5603
Ganglioneuroma (%)	Diagnosis (D)	94	0.07 (0, 1.94E107)	0.9837	0.07 (0, 1.57E115)	0.9848
	Resection (R)		0.99 (0.97, 1.00)	0.0796	0.99 (0.98, 1.01)	0.2222
	(R − D)		0.99 (0.97, 1.00)	0.0858	0.99 (0.98, 1.01)	0.2346
Histology—GNB, inter/nod (%)	Diagnosis (D)	1	1.0 (1.0, 1.0)	ND	1.0 (1.0, 1.0)	ND
	Resection (R)		1.0 (1.0, 1.0)	ND	1.0 (1.0, 1.0)	ND
	(R − D)^**^		1.0 (1.0, 1.0)	ND	1.0 (1.0, 1.0)	ND
Necrosis (%)	Diagnosis (D)	94	1.002 (0.99, 1.01)	0.6299	1.003 (0.99, 1.01)	0.5523
	Resection (R)		1.0 (0.99, 1.01)	0.9372	0.998 (0.99, 1.01)	0.5833
	(R − D)		0.999 (0.99, 1.01)	0.7107	0.997 (0.99, 1.00)	0.3907

^a^ Sample size with known data at both diagnosis and resection.

^b^ Amount of increased risk for every one unit increase of the value of the histologic factor.

* Univariate Cox PH model.

** Denotes *p* < 0.05.

### 3.4 Prognostic Value of Changes in Histology Following Induction Therapy

No shift in histologic factor between diagnosis and tumor resection was prognostic of outcome in a statistically significant manner. For each 1% increase in the change of the percentage of intermediate/high MKI between diagnosis and resection, there was a trend toward a 1% decreased risk of a PFS event (HR = 0.99; 95% CI: 0.99–1.00; *p* = 0.0506) or OS event (HR = 0.99; 95% CI: 0.99–1.00; *p* = 0.0588) (Table 4). Compared to patients with low tumor MKI at both diagnosis and resection, those with intermediate/high tumor MKI at diagnosis and low MKI at resection had 2.1 times and 2.3 times increased risk of a PFS or OS event, respectively, although this did not reach statistical significance (PFS HR = 2.1; 95% CI: 0.9–4.9; *p* = 0.0908), (OS HR = 2.3; 95% CI: 0.9–5.9; *p* = 0.0773) (Table 3).

A key finding from our initial study was that the presence of > 90% persistent tumor cells after induction chemotherapy was associated with a poor prognosis [25]. Assessed in this cohort, the presence of NCs ≥ 90% was not prognostic of PFS or OS (Table 4). Similarly, ≤ 10% necrosis at tumor resection was not prognostic of PFS (*p* = 0.8419) or OS (*p* = 0.5725) (Table 3). While the percentage of tumors with low levels of stroma (stroma‐poor) was significantly decreased from diagnosis to resection, this did not translate to improved prognostic ability for PFS or OS. A decrease in tumors with undifferentiated or poorly differentiated histology also did not exhibit a statistically significant improvement in the ability to predict PFS (Table 4). Therefore, between diagnosis and resection, decreased tumor MKI, the presence of persistent tumor cells, or shift to a more differentiated phenotype was not prognostic of PFS or OS.

### 3.5 Assessment of Outcome Stratified by *MYCN* Status Does Not Lead to Improved Prognostic Ability

*MYCN* amplification may independently predict the risk of tumor progression and confound alternative prognostic factors [24]; while specific prognostic features in patients with high‐risk neuroblastoma without tumor MYCN amplification are unclear. Therefore, we repeated our analyses excluding all tumor samples with *MYCN* amplification. Within the subgroup of patients with *MYCN* non‐amplified neuroblastoma and age ≥ 18 months (*N* = 47), somewhat smaller differences were observed; a ≥ 90% decrease in persistent neuroblastoma cells was seen in 28% (*p* = 0.005) of tumors following induction therapy, while no appreciable alterations in necrosis were seen, in keeping with the nature of tumors without MYCN amplification (Table 5). In this subset, no significantly prognostic histological feature was identified with the hazard ratios in this group being similar to those in the overall cohort. The prognostic ability for OS was not significantly influenced by the change in the percent of NCs or necrosis from diagnosis to resection (*p* = 0.0964 and *p* = 0.1338, respectively) (Table 6). When assessed as categorical variables with a cutoff of ≥ 90% for NCs and ≤ 10% necrosis, neither feature was significantly prognostic (Table 6). Additional cutoff points tested for necrosis or NCs also failed to identify a significant prognostic threshold (Tables S1 and S2).

**TABLE 5:** Histologic factors within the subgroup age ≥ 18 months at diagnosis with *MYCN* non‐amplified tumors at diagnosis and resection, and identification of significant changes from diagnosis to resection (*n* = 47).

Histologic factor (binary)^a^	Timepoint	Sample size *n* (%)	*n* (%)	*p* *
NCs ≥ 90%	Diagnosis (D)	47 (100)	36 (77)	0.005
	Resection (R)		23 (49)	
	(R − D)^b^		−13 (−28)	
Necrosis ≤ 10%	Diagnosis (D)	47 (100)	36 (77)	0.1
	Resection (R)		30 (64)	
	(R − D)^b^		−6 (−13)	

Abbreviation: NCs, neuroblastic cells.

^a^ Histologic factors with binary values were created by combining categories or applying a percentage threshold.

^b^ See Table S1 for crosstabulations by timepoint.

* McNemar's test of paired comparison of diagnosis versus resection.

**TABLE 6:** Prognostic ability of continuous or categorical histologic factors for PFS and OS, at diagnosis, at resection, and the change from diagnosis to resection for tumors with *MYCN* non‐amplification and patient age ≥ 18 months (*n* = 47).

Histologic factor (continuous)						
	Timepoint	*n* ^a^	PFS hazard ratio (95% CI)^b^	PFS *p*‐value*	OS hazard ratio (95% CI)^b^	OS *p*‐value*
NCs ≥ 90% (%)	Diagnosis (D)	47	0.998 (0.99, 1.01)	0.5646	0.995 (0.99, 0.003)	0.2062
	Resection (R)	47	1.001 (0.99, 1.01)	0.8281	1.01 (0.996, 1.01)	0.2897
	(R − D)	47	1.002 (0.996, 1.01)	0.5528	1.01 (0.999, 1.01)	0.0964
Necrosis ≤ 10% (%)	Diagnosis (D)	47	0.998 (0.99, 1.01)	0.5646	0.995 (0.99, 0.003)	0.2062
	Resection (R)	47	0.998 (0.99, 1.01)	0.7018	1.003 (0.99, 1.01)	0.5650
	(R − D)	47	1.001 (0.99, 1.01)	0.8729	1.01 (0.998, 1.01)	0.1338

Histologic factor (categorical)								
	Timepoint	*n*	5‐year PFS ± SE (%)	PFS hazard ratio (95% CI)^b^	PFS *p*‐value*	5‐year OS ± SE (%)	OS hazard ratio (95% CI)^b^	OS *p*‐value*
NCs category		47						
≥ 90%	Resection (R)	23	34 ± 10.0	1.3 (0.6, 2.6)	0.5105	42 ± 10.5	1.5 (0.7, 3.3)	0.2686
< 90% [ref]		24	46 ± 10.2			67 ± 9.6		
Necrosis category		47						
≤ 10%	Resection (R)	30	36 ± 8.9	1.02 (0.5, 3.2)	0.9604	49 ± 9.3	1.4 (0.6, 3.0)	0.4462
> 10% [ref]		17	47 ± 12.1			65 ± 11.6		

Abbreviation: ND, not done.

^a^ Sample size with known data at both diagnosis and resection.

^b^ Amount of increased risk for every one unit increase of the value of the histologic factor.

* Univariate Cox PH model.

## 4 Discussion

Tumors undergo significant histological changes following chemotherapy [25, 30]. However, it remains unclear whether the histologic shift toward a more favorable phenotype is predictive of prognosis and whether it could guide therapy modifications early in treatment. Our initial single‐institution pilot study (*n* = 43) indicated that a progressive increase in necrosis and a reduction in tumor cell percentage at resection correlated with outcome [25]. Expanding on these findings, in the current study, we analyzed paired tissue samples from 94 patients with high‐risk neuroblastoma to quantify histologic changes and evaluate their prognostic value following induction therapy.

The findings revealed a clear evolution in tumor histology following induction chemotherapy, indicated by features suggestive of cellular differentiation or death. Specifically, there was a notable reduction in high or intermediate tumor MKI and an increase in differentiation and necrosis from diagnosis to resection. Other features, such as increased neuropil content supported a trend toward a more differentiated tumor phenotype. While individual unfavorable tumor characteristics significantly shifted toward a more favorable phenotype following induction chemotherapy, none of these histologic changes were independently predictive of outcome. Consistent with prior studies [8, 24, 31], intermediate/high MKI at diagnosis remained a robust predictor of poor overall and progression‐free survival. However, the presence of any single favorable histologic feature at resection, whether analyzed as a continuous or binary variable, was not prognostic. This result is discrepant with the findings from our pilot study [25]. The lack of agreement between studies can be attributed to multiple factors, including an underpowered pilot study to detect prognostic factors that apply to a broader population. To address this limitation, we included more patients in the current study who were treated at multiple sites. Site‐specific variation in diagnostic and treatment practices may also have influenced results.

Several limitations may have influenced our results. The well‐documented cellular and molecular heterogeneity of neuroblastoma, combined with sampling constraints at both diagnosis and resection, likely limited the representativeness of the sampled tumor sections. Differences in sampling practices among providers at different sites may also have led to variation in results as could minor variations in induction chemotherapy regimens across centers and differences in timing of resection (e.g., after 5 vs. 6 cycles of therapy). Importantly, other biological variables such as chromosomal alterations and ALK mutation status were not considered. The limited cohort size in this study may also have constrained our ability to detect nuanced relationships between histological changes and outcomes.

The goal of this study was to identify histologic characteristics predictive of outcome that could be used to justify an individually tailored approach to therapy. Previous studies have demonstrated that end‐of‐induction response, assessed using the International Neuroblastoma Response Criteria [32], significantly influences survival [14]. Factors such as age < 18 months, absence of metastases, MYCN amplification, 1p LOH, absence of 11q LOH, and high MKI have been predictive of early response. The fact that histologic changes following induction therapy are not significant predictors of outcome suggests that changes to risk stratification after induction chemotherapy and subsequent adjustments in therapeutic management should not be based solely on primary tumor histologic characteristics. For one thing, histological analysis does not account for metastatic disease nor does it reflect tumor biology following multimodal therapy exposure such as myeloablative conditioning regimens and immunotherapy. In addition, it is becoming apparent that prognostic factors at diagnosis need to be reassessed continually throughout treatment. Recent results from data analysis of a much larger patient cohort (*N* = 1244), indicate that the impact of prognostic factors at diagnosis was not maintained in patients with varying responses to induction [33]. In this analysis of several conventionally used prognostic features, including INPC histologic categories (favorable vs. unfavorable), MKI (high vs. low or intermediate), and grade of differentiation (differentiating vs. poorly differentiated or undifferentiated), in patients with complete or partial responses to induction, only the absence of stage 4 disease was predictive of a favorable outcome.

Further studies may determine whether histological features at resection correlate with the currently used revised response assessment that integrates tumor response in the primary tumor, soft tissue, bone and bone marrow metastasis [19]. Our future work will aim to include histological profiling together with response metrics such as mIBG scoring and contemporary methods of histological analysis that incorporate artificial intelligence‐based classification [34]. Incorporation of transcriptional and epigenetic profiling of tumor cells at the single‐cell level would elucidate chemotherapy‐induced cellular transitions which could then be used to identify molecular or genetic biomarkers to complement these response assessment methods. Ultimately, despite the dramatic histological shift after induction chemotherapy, the lack of prognostic value of this change suggests that measures of molecular response, such as circulating nucleic acid analysis may be a better modality in the future [35, 36]. We propose that investigators should continue to invest in the study of molecular biomarkers as response metrics, which may be more representative of tumor response than gross histologic changes.

## Author Contributions

**Monica Pomaville:** data curation (lead), formal analysis (equal), investigation (lead), methodology (equal), visualization (lead), writing – original draft (lead), writing – review and editing (equal). **Pei‐Chi Kao:** formal analysis (lead), software (lead), validation (lead), visualization (equal), writing – review and editing (supporting). **Antonio Perez‐Atayde:** data curation (lead), investigation (equal), methodology (equal), writing – review and editing (supporting). **Wendy B. London:** formal analysis (lead), investigation (equal), methodology (equal), project administration (equal), software (equal), supervision (equal), writing – original draft (equal), writing – review and editing (equal). **Rani E. George:** conceptualization (lead), data curation (equal), formal analysis (equal), funding acquisition (lead), investigation (equal), supervision (equal), writing – review and editing (lead).

## Ethics Statement

This study was conducted in accordance with the Declaration of Helsinki. The Dana‐Farber Cancer Institute institutional review board approved this study and granted a waiver of informed consent due to the retrospective nature of the study and use of fully de‐identified patient data (Protocol 13‐039).

## Conflicts of Interest

W.B.L. reports consulting work for Jubilant DraxImage as a member of a Data Safety Monitoring Board.

## Supporting information


**Table S1:** Prognostic ability of continuous histologic factors for PFS and OS, at diagnosis, at resection, and the change from diagnosis to resection for different cutoff points.
**Table S2:** Prognostic ability of categorical histologic factors for PFS and OS, at diagnosis, at resection, and the change from diagnosis to resection for different cutoff points (*n* = 94).

## Data Availability

The data that support the findings of this study are available on request from the corresponding author. The data are not publicly available due to privacy or ethical restrictions.
