# Supplementary material for: Histologic Response to Induction Chemotherapy in High‐Risk Neuroblastoma
Source: Cancer Med. 2025 Nov 26;14(22):e71332. doi: 10.1002/cam4.71332 (PMC12648435; doi:10.1002/cam4.71332)
Supplement: Supplementary file 1 — Table S1: Prognostic ability of continuous histologic factors for PFS and OS, at diagnosis, at resection, and the change from diagnosis to resection for different cutoff points. Table S2: Prognostic ability of categorical histologic factors for PFS and OS, at diagnosis, at resection, and the change from diagnosis to resection for different cutoff points (n = 94). [file CAM4-14-e71332-s001.docx]

Supplementary Tables

Supplementary Table S1. Prognostic ability of continuous histologic factors for PFS and OS, at diagnosis, at resection, and the change from diagnosis to resection for different cutoff points

| Within the subgroup of *MYCN* non-amp and age ≥18 mo (n=47) | | | | | | |
| --- | --- | --- | --- | --- | --- | --- |
| Histologic factor  (continuous) | Timepoint | n^@^ | PFS  Hazard ratio  (95% CI) ^#^ | PFS  p-value* | OS  Hazard ratio  (95% CI) ^#^ | OS  p-value* |
| NC - ≥80 % (%) | Diagnosis (D)  Resection (R)  (R – D) | 47  47  47 | 1.000 (0.99, 1.01)  1.01 (0.996, 1.01)  1.003 (0.996, 1.01) | 0.9964  0.2768  0.4069 | 0.998 (0.99, 1.01)  1.01 (0.996, 1.01)  1.004 (0.997, 1.01) | 0.6897  0.2746  0.2707 |
| NC - ≥70 % (%) | Diagnosis (D)  Resection (R)  (R – D) | 47  47  47 | 1.002 (0.99, 1.01)  1.004 (0.996, 1.01)  1.002 (0.995, 1.01) | 0.6987  0.3176  0.5938 | 1.001 (0.99, 1.01)  1.01 (0.996, 1.01)  1.003 (0.996, 1.01) | 0.8754  0.2954  0.4690 |
| NC - ≥60 % (%) | Diagnosis (D)  Resection (R)  (R – D) | 47  47  47 | 1.003 (0.99, 1.01)  1.006 (0.997, 1.02)  1.002 (0.995, 1.01) | 0.5525  0.1947  0.5286 | 1.003 (0.99, 1.01)  1.01 (0.998, 1.02)  1.003 (0.996, 1.01) | 0.6150  0.1320  0.3919 |
| Necrosis - ≤20% (%) | Diagnosis (D)  Resection (R)  (R – D) | 47  47  47 | 1.000 (0.99, 1.01)  1.001 (0.99, 1.01)  1.001 (0.99, 1.01) | 0.9964  0.7741  0.8107 | 0.998 (0.99, 1.01)  1.002 (0.99, 1.01)  1.003 (0.995, 1.01) | 0.6897  0.6274  0.4661 |
| Necrosis - ≤30% (%) | Diagnosis (D)  Resection (R)  (R – D) | 47  47  47 | 1.002 (0.99, 1.01)  1.000 (0.99, 1.01)  0.998 (0.99, 1.01) | 0.6987  0.9551  0.6949 | 1.001 (0.99, 1.01)  1.001 (0.99, 1.01)  1.000 (0.99, 1.01) | 0.8754  0.7945  0.9173 |
| Necrosis - ≤40% (%) | Diagnosis (D)  Resection (R)  (R – D) | 47  47  47 | 1.003 (0.99, 1.01)  1.001 (0.99, 1.01)  0.999 (0.99, 1.01) | 0.5525  0.8314  0.7422 | 1.003 (0.99, 1.01)  1.003 (0.99, 1.01)  1.000 (0.99, 1.01) | 0.6150  0.5984  0.9581 |

^@^Sample size with known data at both diagnosis and resection; * univariate Cox PH model; ^#^ amount of increased risk for every one unit increase of the value of the histologic factor

Supplementary Table S2. Prognostic ability of categorical histologic factors for PFS and OS, at diagnosis, at resection, and the change from diagnosis to resection for different cutoff points (n=94)

| Histologic factor  (categorical) | Timepoint | n | 5-year  PFS ±SE  (%) | PFS  Hazard ratio (95% CI)^#^ | PFS  p-value* | 5-year  OS ±SE  (%) | OS  Hazard ratio (95% CI) ^#^ | OS  p-value* |
| --- | --- | --- | --- | --- | --- | --- | --- | --- |
| NCs category |  | 94 |  |  |  |  |  |  |
| ≥ 80%  < 80% [ref] | Resection (R) | 45  49 | 28 ± 6.8  45 ± 7.1 | 1.4 (0.9, 2.3) | 0.1842 | 38 ± 7.4  59 ± 7.1 | 1.5 (0.9, 2.6) | 0.1264 |
| ≥ 70%  < 70% [ref] | Resection (R) | 55  39 | 28 ± 6.2  49 ± 8.0 | 1.5 (0.9, 2.6) | 0.1078 | 40 ± 6.7  61 ± 7.8 | 1.4 (0.8, 2.4) | 0.2301 |
| ≥ 60%  < 60% [ref] | Resection (R) | 56  38 | 28 ± 6.1  50 ± 8.1 | 1.6 (0.9, 2.6) | 0.0966 | 39 ± 6.7  63 ± 7.9 | 1.5 (0.8, 2.5) | 0.1718 |
| Necrosis category |  | 94 |  |  |  |  |  |  |
| ≤20%  >20% [ref] | Resection (R) | 54  40 | 33 ± 6.5  43 ± 7.3 | 1.2 (0.7, 2.0) | 0.4938 | 43 ± 6.9  57 ± 7.9 | 1.4 (0.8, 2.3) | 0.2734 |
| ≤30%  >30% [ref] | Resection (R) | 64  30 | 32 ± 5.9  47 ± 9.1 | 1.3 (0.8, 2.3) | 0.3288 | 44 ± 6.3  60 ± 9.0 | 1.3 (0.7, 2.3) | 0.3668 |
| ≤40%  >40% [ref] | Resection (R) | 66  28 | 31 ± 5.8  50 ± 9.5 | 1.4 (0.8, 2.5) | 0.2427 | 44 ± 6.2  61 ± 9.3 | 1.5 (0.8, 2.7) | 0.2274 |
| Within the subgroup of *MYCN* non-amp and age ≥18 mo (n=47) | | | | | | | | |
| Histologic factor  (categorical) | Timepoint | n | 5-year  PFS ±SE  (%) | PFS  Hazard ratio (95% CI)^#^ | PFS  p-value* | 5-year  OS ±SE  (%) | OS  Hazard ratio (95% CI) ^#^ | OS  p-value* |
| NCs category |  | 47 |  |  |  |  |  |  |
| ≥ 80%  < 80% [ref] | Resection (R) | 27  20 | 29 ± 8.9  55 ± 11.1 | 1.6 (0.8, 3.5) | 0.1937 | 47 ± 9.8  65 ± 10.7 | 1.6 (0.7, 3.5) | 0.2451 |
| ≥ 70%  < 70% [ref] | Resection (R) | 31  16 | 31 ± 8.5  56 ± 12.4 | 1.4 (0.6, 3.1) | 0.4074 | 47 ± 9.1  69 ± 11.6 | 1.5 (0.7, 3.4) | 0.3350 |
| ≥ 60%  < 60% [ref] | Resection (R) | 32  15 | 30 ± 8.3  60 ± 12.7 | 1.5 (0.7, 3.3) | 0.3484 | 46 ± 8.9  73 ± 11.4 | 1.7 (0.7, 4.1) | 0.2095 |
| Necrosis category |  | 47 |  |  |  |  |  |  |
| ≤20%  >20% [ref] | Resection (R) | 33  14 | 33 ± 8.3  57 ± 13.2 | 1.3 (0.6, 3.0) | 0.4798 | 51 ± 8.8  64 ± 12.8 | 1.4 (0.6, 3.3) | 0.4547 |
| ≤30%  >30% [ref] | Resection (R) | 36  11 | 36 ± 8.1  55 ± 15.0 | 1.02 (0.4, 2.4) | 0.9646 | 52 ± 8.4  64 ± 14.5 | 1.2 (0.5, 3.1) | 0.6412 |
| ≤40%  >40% [ref] | Resection (R) | 36  11 | 36 ± 8.1  55 ± 15.0 | 1.02 (0.4, 2.4) | 0.9646 | 52 ± 8.4  64 ± 14.5 | 1.2 (0.5, 3.1) | 0.6412 |

* univariate Cox PH model

^#^ amount of increased risk for every one unit increase of the value of the histologic factor

ND – not done
